# The Exceedingly Rapid Development of an Intracranial Aneurysm

**DOI:** 10.7759/cureus.32636

**Published:** 2022-12-17

**Authors:** Francesco Massari

**Affiliations:** 1 Radiology, University of Massachusetts Medical School, Worcester, USA

**Keywords:** de-novo, brain, coiling, sah, aneurysm

## Abstract

Despite significant diagnostic and technical progress in managing intracranial aneurysms, there are still open questions in understanding their pathophysiology: how fast can they form and grow? We had the chance to observe the "de novo" genesis and rupture of an aneurysm of a left MCA posterior trunk M3 branch within 14 days in one of our patients. We were in the position to compare an initially inconspicuous vessel, assessed during a diagnostic cerebral angiogram with 3D acquisitions, performed as an elective follow-up to monitor the decade stability of a transitional aneurysm in the same vascular territory, and the same vessel only two weeks after, harboring a new small ruptured aneurysm. Several studies along the intracranial aneurysms' pathophysiology have been reported but primarily oriented toward identifying uncommon conditions such as inherent defects in collagen synthesis, genetic or familial factors, or basic anatomic variations or abnormalities in the cerebral vasculature. Suppose this case report does not pretend to provide a clear answer to these questions. However, it is up to date, the shortest time (14 days) reported in the literature for a well-documented "de novo" genesis and rupture of an intracranial aneurysm "in vivo" in humans. The purpose of this case report is not only to underscore the unpredictability of this vascular disease but, even more, to support the idea that further investigation, with more modern methodologies, is of paramount importance in determining the etiopathogenesis and behavior of this stealthy disease.

## Introduction

Intracranial aneurysms are vascular lesions, primarily acquired in etiology, mainly centered at the branching points of the major arterial vessels [1]. Intracranial aneurysms (IA) are found in approximately 2% of the general population [2]. Most intracranial aneurysms (80 to 85%) are located in the anterior circulation, most commonly at the junction of the internal carotid artery and the posterior communicating artery, the anterior communicating-artery complex, or the bifurcation/trifurcation of the middle cerebral artery [3]. Aneurysms of the posterior circulation are most frequently located at the bifurcation of the basilar artery or the junction of a vertebral artery and the ipsilateral posterior inferior cerebellar artery [3-4]. Multiple intracranial aneurysms, usually two or three in number, are found in 20 to 30 percent of patients [3-5].

Ruptured intracranial aneurysms are the most common cause of non-traumatic subarachnoid hemorrhage (SAH), with an annual incidence of approximately 1 per 10,000 people, accounting for approximately 28,000 deaths each year in North America [6-8]. An aneurysmal subarachnoid hemorrhage is a devastating event associated with high morbidity and mortality rates. Approximately 12% of patients perish before receiving medical attention. Although medical and surgical management has significantly improved in recent years, the one-month death rate in hospitalized patients is still around 40%. More than one-third of the survivors present major neurologic deficits, and there are some cognitive deficit sequelae in many patients with overall good outcomes [9].

Despite significant diagnostic and technical progress in managing intracranial aneurysms, there are still open questions in understanding their pathophysiology: how fast can they form and grow? We had the chance to observe the "de novo" genesis and rupture of an aneurysm of a left MCA posterior trunk M3 branch within 14 days in one of our patients. The purpose of this case report is not only to underscore the unpredictability of this vascular disease but, even more, to support the idea that further investigation, with more modern methodologies, is of paramount importance in determining the etiopathogenesis and behavior of this stealthy disease.

## Case presentation

**Figure 1:**
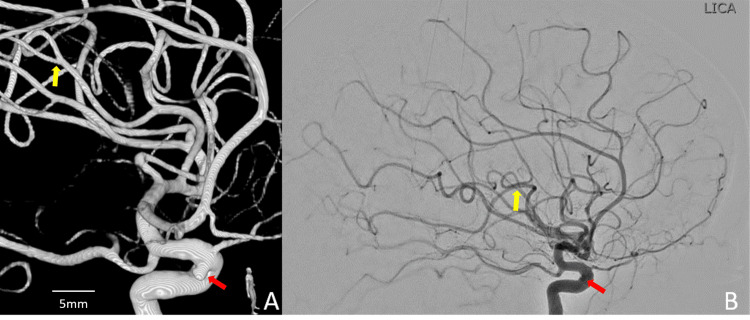
3D and Angiographic images (September 2020) 3D (A) and Angiographic (B) images (September 2020), confirming the stability of the left ICA cavernous/cave 2.7 mm aneurysm (red arrow) in comparison with the cerebral angiogram performed in 2011. No new aneurysm was noted at the level of the left anterior circulation (yellow arrow, used as a reference to anticipate the location of the ruptured left MCA posterior trunk M3 bifurcation aneurysm).

A patient is their late 50s was initially found to have an unruptured 2 mm cavernous/cave left internal carotid artery (ICA) aneurysm during a brain MRA performed in 2010 as work-up for migraine headache, confirmed in a 2011 cerebral angiogram. Regarding the size and location aneurysm, endovascular treatment wasn't deemed indicated. Two follow-up head CTA, the most recent in the 3rd quarter of 2020, demonstrated a stable left ICA aneurysm. An elective follow-up cerebral angiogram, performed at the end of September 2020, demonstrated an unchanged left ICA aneurysm. No new aneurysms were observed. A cranial 3-D rotational angiography acquisition of the left anterior circulation was performed to accurately determine changes in the left ICA aneurysm size or shape compared with the 2011 angiographic study (Figure 1).

**Figure 2:**
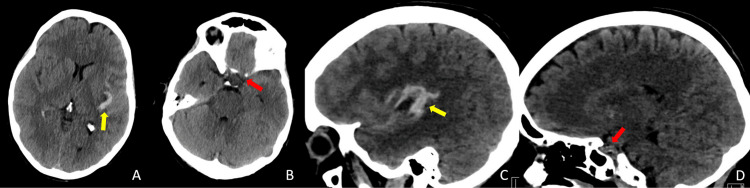
Emergent Head CT (October 2020) Emergent Head CT (October 2020) indicative of an acute subarachnoid hemorrhage involving the left Sylvian fissure extending to the left frontal and temporal sulci (A axial and C sagittal images). No hemorrhagic extension within the basal cisterns was noted (B axial and D sagittal images)—yellow arrow indicating the SAH distribution. The red arrow was used as a reference to define where they would have been located the SAH in case of the left ICA aneurysm rupture.

The patient presented to the Emergency Department of our University Hospital in early October 2020 (14 days post-cerebral angiography) with a 10/10 headache that began in the morning. The patient stated that he was in his usual state of health until they suddenly experienced an abrupt onset of left-sided headache involving the eye, forehead, entire left-sided scalp, and neck. The patient described this as "the worst headache of my life." The patient denied fever or chills. The patient self-administered Tylenol, which is generally helpful in controlling its usual migraine symptoms; however, this provided no pain relief. No focal neurological symptoms were observed. The patient tested negative for COVID-19. A subsequent head CT indicated an acute subarachnoid hemorrhage involving the left Sylvian fissure extending to the left frontal and temporal sulci with no basal cisterns extension (Figure 2).

**Figure 3:**
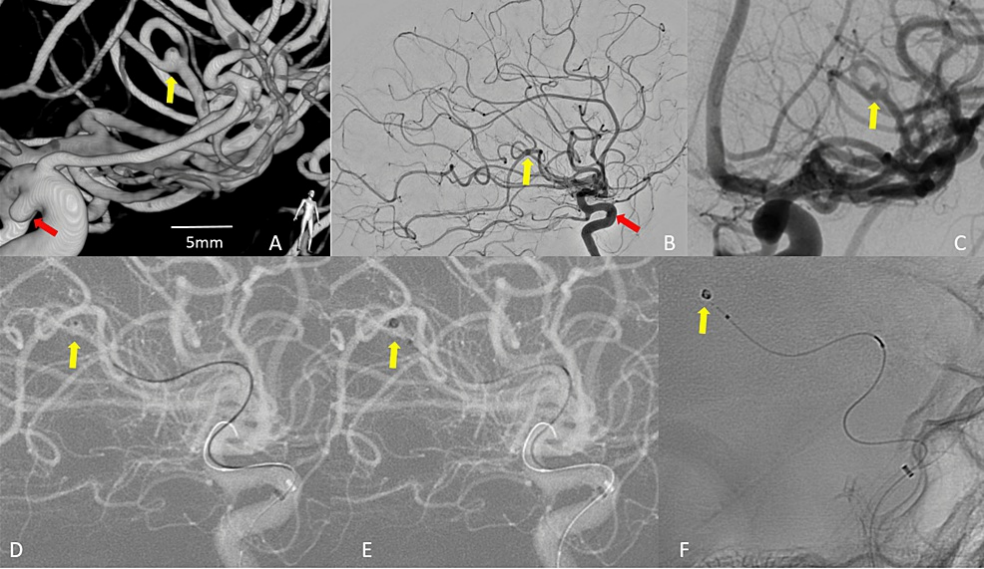
Emergent angiographic images Emergent angiographic images further confirm the stability in size and shape of the LICA cavernous/cave aneurysm (red arrow). New 1.6 x 1.3 mm left MCA posterior trunk M3 bifurcation aneurysm (A, B) (yellow arrow). Furthermore, a small eccentric clot is sitting within the superior branch of the left MCA M3 bifurcation (C). Successful primary coil embolization of the ruptured left MCA aneurysm was achieved with a 1 mm coil (D, E, F) (yellow arrow).

The patient was then transferred to the Angio-Lab for a cerebral angiogram with the intent of endovascularly treating the known left ICA aneurysm. The angiogram of the left ICA further demonstrated stability in size and shape of the LICA cavernous/cave aneurysm, with no evidence of dome pseudolobules concerning for rupture point. Unexpectedly, a thorough analysis of the entire left ICA vascular tree indicated a new 1.6 x 1.3 mm left middle cerebral artery (MCA) posterior trunk M3 bifurcation aneurysm, likely representing about the rapid growth, an undetermined etiology aneurysm. Furthermore, a small clot component was observed sitting eccentrically within the superior branch of the left MCA posterior trunk M3 segment bifurcation. A successful coil embolization of the ruptured left MCA aneurysm was achieved with a 1 mm coil (Figure 3). The patient underwent an uneventful post-endovascular treatment recovery, with no development of focal neurologic symptoms in the three weeks post-aneurysmal rupture. No new SAH or significant cerebral vasospasm has been encountered.

**Figure 4:**
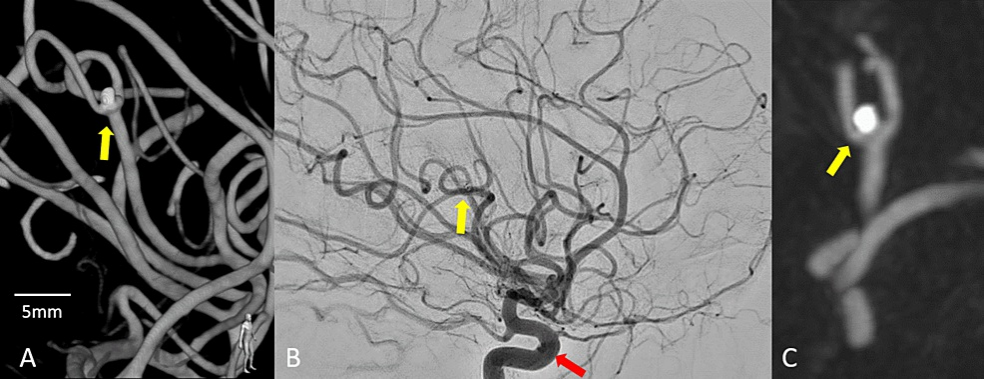
12-day post-procedural cerebral angiogram 12-day post-procedural cerebral angiogram confirming complete coil embolization of the ruptured left MCA posterior trunk M3 bifurcation aneurysm (yellow arrow), with unremarkable patency of the parent arteries. The round left ICA transitional aneurysm (red arrow), again unchanged in size and morphology, compared with the previous three Cerebral Angiograms, the most remote in 2011. No new aneurysms were identified. (A 3D images; B angiographic images; C Vaso-CT images).

**Figure 5:**
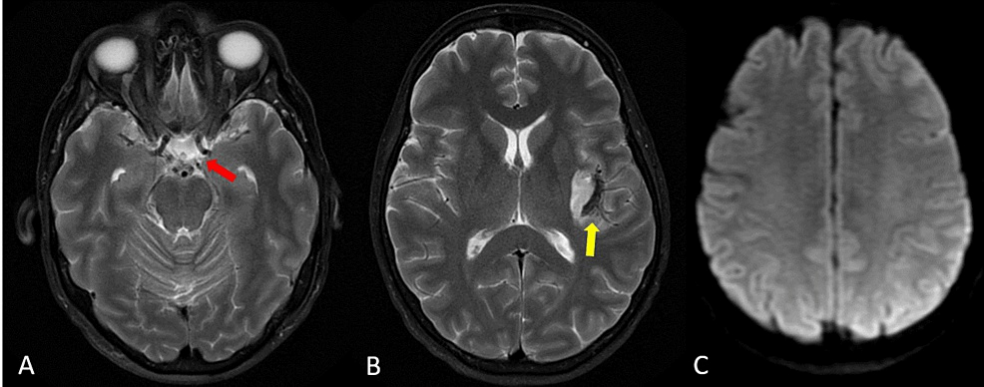
Follow-up Brain MRI Follow-up Brain MRI confirming small residual subarachnoid hemorrhage component within the left Sylvian fissure with no involvement of the basal cisterns (A, B). No procedure-related DWI+ findings within the left MCA posterior trunk vascular distribution (C). Yellow arrow indicating the SAH distribution. The red arrow was used as a reference to define where they would have been located the SAH in case of the left ICA aneurysm rupture.

A 12-day follow-up cerebral angiogram confirmed a complete coil embolization of the ruptured left MCA posterior trunk M3 bifurcation aneurysm, with unremarkable patency of the parent arteries. The round left ICA transitional aneurysm was again unchanged in size and morphology, compared with the previous three cerebral angiograms, the most remote in 2011 (Figure 4). No new aneurysms were identified. The patient has been discharged home 18 days post-aneurysmal rupture after obtaining a follow-up Brain MRI (Figure 5) at baseline clinical status.

**Figure 6:**
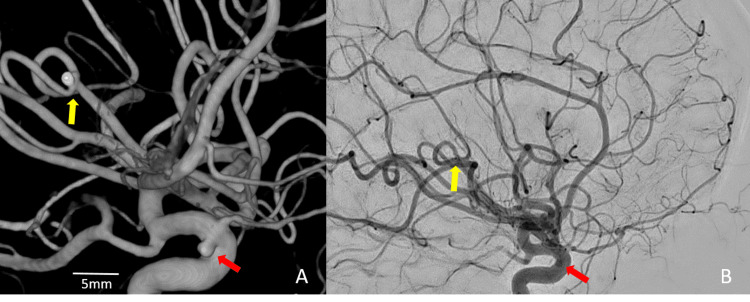
6-month follow-up cerebral angiogram 6-month follow-up cerebral angiogram demonstrating stable complete coil embolization of the ruptured left MCA posterior trunk M3 bifurcation aneurysm (yellow arrow). Unchanged the round left ICA transitional aneurysm (red arrow). No new aneurysms were identified. (A 3D images; B angiographic images).

A 6-month (Figure 6) follow-up cerebral angiogram demonstrated stable complete embolization of the ruptured M3 left MCA aneurysm and unchanged left ICA transitional aneurysm. The stability of the findings were confirmed at one and 2-year follow-up cerebral angiographies.

## Discussion

Intracranial aneurysms are vascular lesions primarily centered at the branching points of the major arterial vessels [3]. An aneurysmal subarachnoid hemorrhage is a devastating event associated with high morbidity and mortality rates [3]. Intracranial aneurysms are significantly more common than those originating from extracranial arteries of similar caliber [1]. One possible explanation for this discrepancy resides in the attenuated tunica media and lack of external elastic lamina of the intracranial arteries. On microscopical examination, the typical saccular aneurysm has a very thin tunica media or none, and the internal elastic lamina is either absent or severely fragmented. Thus, the aneurysm wall comprises only intima and adventitia, with variable fibrohyaline tissue interposed between these two layers [2].

The reason the trends in morbidity and mortality from ruptured berry aneurysms have remained mostly the same over the past several decades [10-14] is primarily related to the fact that the diagnosis of berry aneurysms is generally made only after the rupture has occurred. This is mainly caused by the frequently vague and nonspecific neurologic symptoms associated with the impending rupture of a berry aneurysm and the elevated incidence of headaches in the otherwise healthy general population.

The main problem associated with intracranial berry aneurysms is the lack of definitive predicting factors in their development. Several studies on this topic have been reported. However, they were oriented mainly toward identifying relatively uncommon conditions such as inherent defects in collagen synthesis [15], genetic or familial factors [16-17], or basic anatomic variations or abnormalities in the cerebral vasculature [18-20]. 

If the combination of genetic factors (such as the association of intracranial aneurysms with heritable connective-tissue disorders and familial occurrence [21-22]) and environmental factors (among others, the rare incidence in children, the mean age of patients with aneurysmal subarachnoid hemorrhage around 50 years, the association of aneurysmal subarachnoid hemorrhage risk with cigarette smoking [23], hypertension [24], moderate-to-high level of alcohol consumption and hypercholesterolemia), would be at the base of aneurysm genesis; how fast an aneurysm forms and its rate of expansion are still unanswered questions.

## Conclusions

Suppose this case report does not pretend to provide a clear answer to these questions. However, it is up to date, the shortest time (14 days) reported in the literature for a well-documented "de novo" genesis and rupture of an intracranial aneurysm "in vivo" in humans. We were in the position to compare an initially inconspicuous vessel, assessed during a diagnostic cerebral angiogram with 3D acquisitions, performed as an elective follow-up to monitor the decade stability of a transitional aneurysm in the same vascular territory, and the same vessel only two weeks after, harboring a new small ruptured aneurysm. The purpose of this case report is not only to underscore the unpredictability of the aneurysmatic vascular disease but, even more, to support the idea that further investigation, with more modern methodologies, is of paramount importance in determining the determination of the etiopathogenesis and behavior of this stealthy disease.
